# Supplementary material for: Human Colorectal Cancer Infrastructure Constructed by the Glycocalyx
Source: J Clin Med. 2019 Aug 22;8(9):1270. doi: 10.3390/jcm8091270 (PMC6780787; doi:10.3390/jcm8091270)
Supplement: Supplementary file 1 [file jcm-08-01270-s001.pdf]

Table S1. Characteristics of CRC patients with surgical operation.

Case number	Age (yr)	Sex	primary location	Size (cm)	histology	TNM Stage	T	N	M	previous chemotherapy	previous other treatment
1	67	male	Upper retum	6.6×2.9	Moderately～poorly dfferentiated tubular adenocarcinoma	ⅢC	3	2	0	None	None
2	69	male	Descending colon	2.0×1.8	Moderately～well dfferentiated tubular adenocarcinoma	ⅢC	3	2	0	None	None
3	49	male	Ascending colon	4.5×3.3	Moderately dfferentiated tubular adenocarcinoma	Ⅰ	2	0	0	None	None
4	85	male	Cecum colon	4.0×3.0	papillay ～ well differentiated tubular adenocarcinoma	Ⅰ	2	0	0	None	None
5	51	female	Upper retum	3.0×2.8	mucinous～papillary adenocarcinoma	ⅡA	3	0	0	None	None

Table 2. Chracteristics of lectins in tissues from human CRC patients.

			Expression of Normal tissues		Expression of cancer tissues	
Lectin abbriviation	Source of lectin	Specificity	endothelial cell	epithelial cell	endothelial cell	cancer cell
UEA1	Ulex Europaeus Agglutinin I	Fucose	yes	yes	yes	yes
VVL	Vicia Villosa	GalNAc	no	yes	no	yes
MPL	Maclura pomifera	GalNAc	yes	no	yes	no
Tomato lectin	Lycopersicon esculentum	βGlcNAc	yes	no	yes	no
Potato lectin	Solanum tuberosum	βGlcNAc	yes	no	yes	no

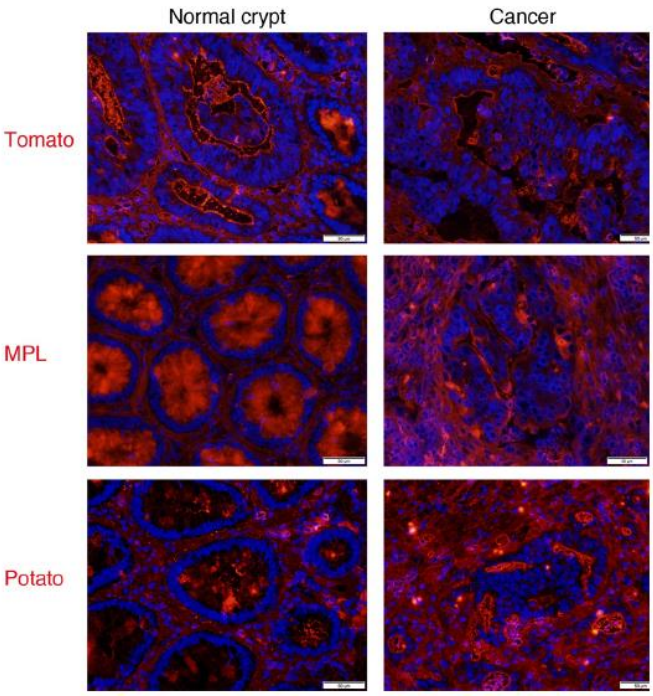

Supplementary Figure S1. Expression of lectins in human CRC tissues. Expression of lectins (Tomato, MPL, and Potato: Red) binding to the GCX in normal and cancerous tissue samples from human patients. Cell nuclei are stained with DAPI (blue). Scale bars: 50 μm. (See also Table S2).

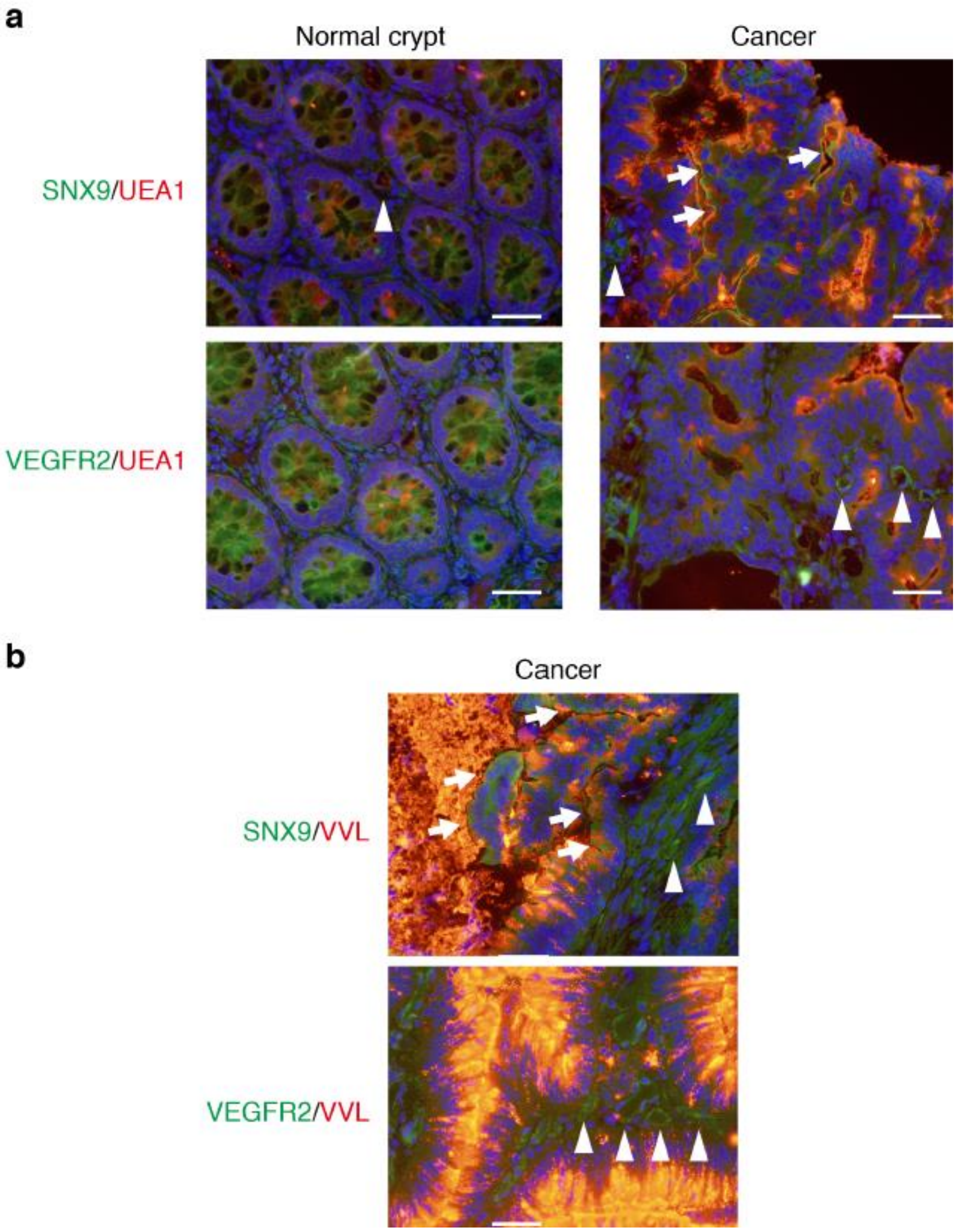

Figure S2. Expression of SNX9 and VEGFR2 expressions in human CRC tissues. (**a**) Co-staining of IF for SNX9/UEA1 and VEGFR2/UEA1 expressions in normal and cancer intercellular regions of human samples. (**b**) Co-staining of IF for SNX9/VVL and VEGFR2/VVL expressions in cancer intercellular regions of human samples. Arrows indicate SNX9-positive cancer cells. Arrowheads indicate VEGFR2-positive vessels. Scale bars: 50 μm.
